# P-1544. Outcomes of *Multi-Drug Resistant Acinetobacter Baumannii (MDR-AB)* Management in a Tertiary Care Hospital in Saudi Arabia: Retrospective Observational Study

**DOI:** 10.1093/ofid/ofae631.1711

**Published:** 2025-01-29

**Authors:** Zahrah H Aljuzair, Reem A AlMahasnah, Afrah Sait, Manal Atta

**Affiliations:** Eastern Health Cluster, Dammam, Ash Sharqiyah, Saudi Arabia; King Fahad Specialist Hospital, Dammam, Ash Sharqiyah, Saudi Arabia; King Fahad Specialist Hospital, Dammam, Ash Sharqiyah, Saudi Arabia; King Fahad Specialist Hospital, Dammam, Ash Sharqiyah, Saudi Arabia

## Abstract

**Background:**

Nosocomial infections caused by multi-drug resistant Acinetobacter baumannii (MDR-AB) are widespread and have been linked to numerous infections, resulting in high rates of morbidity and mortality globally. It has a mortality rate of 17.5%, a resistance rate of 74% to carbapenems, and has been found primarily in immunocompromised patients in Saudi Arabia. There is a lack of a defined treatment standard of care with a limited number of alternatives. To gain additional insight into managing MDR-AB, this study aimed to evaluate the clinical efficacy of various antibiotic regimens and compare the outcomes of monotherapy with those of antibiotic combinations

**Methods:**

A five-years (2018 to 2023) retrospective observational study was conducted at a tertiary care hospital in Saudi Arabia. All patients with positive Acinetobacter baumannii culture were screened. Adult patients ( ≥ 18 years ) with MDR-AB isolated and had received a minimum of 72 hours of antibiotic treatment were included. The primary end point was clinical improvement. Adverse drug events and the 30-day overall mortality rate were evaluated as secondary end points

**Results:**

One hundred patients met the study inclusion criteria. Of those, 45% were oncology or transplant patients and 80% were admitted to the intensive care unit. MDR-AB was associated with several infectious syndromes, including 44% pneumonia, 42% bacteremia, and 13% urinary tract infections. 30% of patients were given meropenem as an empiric treatment, with varying dosage regimens; 26% were given piperacillin/tazobactam. Based on culture sensitivity, 68% of the patients were treated with tigecycline, while 28% were retained on meropenem, despite the fact that 98% of the isolates were carbapenem-resistant. Antibiotic monotherapy has been given only to 39% of patients. Clinical improvement was reported in 66% of the patients with 80% achieved complete improvement. The 30-day mortality rate was 50%. Adverse drug events were rare, with only 3% GI symptoms

**Conclusion:**

MDR-AB in our patients is mainly causing two infectious syndromes in 86%. A combination of antibiotics was used in 61%. High clinical improvement with a simultaneous high 30-day mortality rate highlighted the need for standardized guidelines for the management of MDR-AB infections

**Disclosures:**

**All Authors**: No reported disclosures

